# Comparison of [^18^F]Flutemetamol and [^11^C]Pittsburgh Compound-B in cognitively normal young, cognitively normal elderly, and Alzheimer's disease dementia individuals

**DOI:** 10.1016/j.nicl.2017.08.011

**Published:** 2017-08-14

**Authors:** Val J. Lowe, Emily Lundt, David Knopman, Matthew L. Senjem, Jeffrey L. Gunter, Christopher G. Schwarz, Bradley J. Kemp, Clifford R. Jack, Ronald C. Petersen

**Affiliations:** a Department of Radiology, Mayo Clinic, Rochester, MN, United States; b Department of Health Sciences Research, Mayo Clinic, Rochester, MN, United States; c Department of Neurology, Mayo Clinic, Rochester, MN, United States; d Department of Information Technology, Mayo Clinic, Rochester, MN, United States

## Abstract

**Background:**

Understanding the variation in uptake between different amyloid PET tracers is important to appropriately interpret data using different amyloid tracers. Therefore, we compared the uptake differences in [^18^F]Flutemetamol (FMT) and [^11^C]PiB (PiB) PET in the same people.

**Methods:**

Structural MRI, FMT PET and PiB PET were each performed in 30 young cognitively normal (yCN), 31 elderly cognitively normal (eCN) and 21 Alzheimer's disease dementia (AD) participants. PiB and FMT images for each participant were compared quantitatively using voxel- and region-based analyses. Region of interest (ROI) analyses included comparisons of grey matter (GM) regions as well as white matter (WM) regions. Regional comparisons of each tracer between different groups and comparisons of the two modalities within the different groups were performed. To compare mean SUVr between modalities, and between diagnostic groups, we used paired *t*-tests and Student's *t*-test, respectively. We also compared the ability of the two tracers to discriminate between diagnostic groups using AUROC estimates. The effect of using different normalization regions on SUVr values was also evaluated.

**Results:**

Both FMT and PiB showed greater uptake throughout GM structures in AD vs. eCN or yCN. In all dual-modality group comparisons (FMT vs. PiB in yCN, eCN, and AD), greater WM uptake was seen with FMT vs. PiB. In yCN and eCN greater diffuse GM uptake was seen with FMT vs. PiB. When comparing yCN to eCN within each tracer, greater WM uptake was seen in eCN vs yCN.

**Conclusions:**

Flutemetamol and PiB show similar topographical GM uptake in AD and CN participants and the tracers show comparable group discrimination. Greater WM accumulation with FMT suggests that quantitative differences vs. PiB will be apparent when using WM or GM as a reference region. Both imaging tracers demonstrate increased WM uptake in older people. These findings suggest that using different amyloid tracers or different methods of analyses in serial brain imaging in an individual may result in artifactual amyloid change measurements. Clinical use of several amyloid tracers in the same patient will have challenges that need to be carefully considered.

## Highlights

• White matter uptake is quantitatively elevated in elderly vs. younger control subjects in both FMT and PiB PET scans.
• Quantitatively greater white matter uptake is seen in FMT vs. PiB in Alzheimer’s disease dementia and control subjects.
• Variations between FMT and PiB may create the false appearance of changing brain amyloid levels on serial imaging.
• FMT and PiB have comparable ability to categorize Alzheimer’s disease dementia and control subjects cross-sectionally.
• Methods to cross-calibrate different amyloid PET tracers will need to be validated in serial imaging paradigms.

## 1 Introduction

Positron emission tomography (PET) provides important biomarker information that aids in understanding Alzheimer's disease dementia (AD) and the AD spectrum. PET amyloid imaging with [^11^C]Pittsburgh compound B (PiB) and other PET ligands are used to infer the presence of amyloid-β (Aβ) in the brain (Clark et al., 2012, Driscoll et al., 2012, Johnson et al., 2007, Kantarci et al., 2012, Klunk et al., 2004). PiB-PET is only used for research purposes while several other amyloid PET ligands are used clinically and for research. The National Institute on Aging-Alzheimer's Association (NIA-AA) clinical diagnostic guidelines now include amyloid PET biomarker data as an integral component of the diagnostic algorithm (Albert et al., 2011, McKhann et al., 2011). Given the importance of amyloid status in the study of dementia and in the early diagnosis of probable AD, understanding the differences in various amyloid PET tracers is important.

Prior studies have demonstrated similar diagnostic performance with [^18^F]Flutemetamol (FMT) (GE Healthcare, Inc.) and PiB between AD and normal groups using visual analyses and quantitative comparisons (Mountz et al., 2015, Hatashita et al., 2014). Using cerebellar grey matter (GM) for normalization in quantitative analysis, FMT and PiB standardized uptake value ratios (SUVr) have been shown to be highly correlated (Hatashita et al., 2014). Even though these compounds are very similar in molecular structure, higher cortical SUVr in PiB vs. FMT in AD has also been reported (Hatashita et al., 2014). Higher nonspecific binding of FMT to white matter (WM) vs. PiB has been described as a visual observation (Hatashita et al., 2014) but quantitative confirmation of this finding has not been reported.

Regional amyloid tracer quantification using SUVr on delayed imaging with cerebellar normalization was shown to mimic closely the regional uptake (DVR) determined for PiB-PET by dynamic imaging and has been a common method for cortical normalization of PiB-PET data (Price et al., 2005). Most large studies of amyloid status using PiB-PET have therefore used delayed imaging with reference regions for quantification. We and others have proposed that white matter regions can also be used for normalization with improved quantitative stability in longitudinal amyloid PET imaging (Schwarz et al., 2017, Landau et al., 2015, Chen et al., 2015). While amyloid PET data normalization region selection has been a subject of some attention, no prior reports have quantified the differences between FMT and PiB in typical normalization regions. Possible differences in WM uptake between PET amyloid ligands may impact the comparative results of different tracers when analyzed with WM for normalization. We hypothesized that variations in uptake patterns in GM and WM in different patient groups and between FMT and PiB in the same people, could influence SUVr calculations. Therefore, we performed FMT and PiB PET imaging in the identical participants in 3 participant groups to compare quantitative uptake differences in WM and GM. We evaluated 1) if GM or WM uptake differed quantitatively between FMT and PiB in individuals, 2) how any uptake differences affected SUVr calculations and 3) what the implications of any variations would be in group-wise characterization of young cognitively normal (yCN), elderly cognitively normal (eCN), and AD participant groups.

## 2 Materials and methods

### 2.1 Participant information

Participants were drawn prospectively from the Mayo Clinic Study of Aging (MCSA) as described previously (Jack et al., 2009) or from volunteers responding to study advertisement. This study was approved by the Mayo Clinic and Olmsted Medical Center Institutional Review Boards and all subjects signed written informed consent. The clinical trial registration number with www.clinicaltrials.gov is 12–000118. PiB-PET and MRI scans were performed with the MRI performed a median of 16 days prior to the PET scan. The average number of days between clinical exam and PIB-PET imaging is 42 (Median = 11, IQR: 2,29, Range: 0, 272). Amyloid PET imaging was performed under IND #77924. We designed the trial to recruit groups of 30 to show equivalency of the exams if both C11 PiB and F18 Flutemetamol demonstrate sensitivity and specificity with a lower bound of the 95% confidence interval that exceeds 70% (sensitivity and specificity are below 70%). The FMT and PiB scans were performed within a median of 2 days. A total of 82 participants including 30 yCN (ages > 60), 31 eCN (ages 30–60) and 21 probable AD (based on a consensus diagnosis that includes quantitative data from a brief mental status examination, 9 neuropsychological tests and the Clinical Dementia Rating Scale, as well as clinical and cognitive assessment by neurologists, geriatricians, neuropsychologists, and study nurses (Roberts et al., 2008)) were available for analysis. No adverse events were seen from imaging.

### 2.2 Imaging methods

Amyloid-PET imaging was performed using PiB as previously described with a 40 min uptake delay (Lowe et al., 2014). FMT imaging was performed after injection of 370 MBq (range 333–407 MBq) FMT and a delay of 80 min (Nelissen et al., 2009). Imaging acquisitions were 20 min for both tracers on PET/CT (GE Healthcare, DRX or DRXT). We used SPM5 (Ashburner and Friston, 2005) to co-register PET to MRI, and used unified segmentation on the MRI to produce spatial normalization parameters and a labeled atlas in native subject MRI space for each participant. Regional analysis was performed using the AAL atlas and region sharpening was performed by assigning voxels to GM or WM depending on the SPM5 unified segmentation probabilistic likelihood of each being the true structure based on T1 MRI signal. Global cortical GM PiB or FMT SUVr was computed as the median uptake of voxels in the prefrontal, orbitofrontal, parietal, temporal, anterior cingulate, and posterior cingulate/precuneus regions of interest (ROIs) for each subject normalized to the median uptake in cerebellar crus GM. Specifically, crus 1 and 2 GM regions bilaterally, were used for region normalization with the intent to minimize the contribution of WM signal or potential bleed-in from basal occipital cortex in the normalization region as previously described (Jack et al., 2017) (see Inline Supplementary Fig. 1a). Additionally, global cortical GM from PiB or FMT uptake was normalized to the median uptake in a composite ROI made up of a voxel-weighted average of periventricular (PV) frontal WM, PV occipital WM, PV parietal WM, PV temporal WM and whole cerebellum GM + WM as determined by SPM5 segmentation (described above) and by using visual parcellation of subcortical and periventricular WM by a trained neuro-anatomist as previously reported (Murray et al., 2010). This composite ROI is similar to another recently described composite ROI (Landau et al., 2015) although the present composite does not include subcortical WM (Inline Supplementary Fig. 1b). Regional, individual WM SUVr values were also computed using PV frontal, occipital, parietal, or temporal WM regions with cerebellar crus for normalization.

Partial volume correction (PVC), using the 2-compartment model, was used in all calculations (Meltzer et al., 1999). To further assess the effect of white matter bleed-in, we used 3-compartment PVC, that additionally corrects for grey matter and white matter partial volume averaging using a procedure previously described by Muller-Gartner et al. (1992). MRIs were performed at 3 Tesla using an 8-channel phased array coil (GE, Milwaukee, WI). Image acquisition and image analysis are described in detail elsewhere (Lowe et al., 2009).

### 2.3 Image and statistical analysis

PIB and FMT images were normalized to custom template space using the normalization parameters for their co-registered MRI scans, as computed with SPM5 unified segmentation. Paired *t*-test analyses in SPM5 were performed, where each pair consisted of the spatially normalized, cerebellar crus scaled PiB and FMT images for each participant, allowing direct assessment of the differences between the two ligands. Results were false discovery rate (FDR) corrected at *p* < 0.05 and no regions masking was performed. We also conducted ROI-wise analyses to examine differences between the two ligands by atlas region. ROI analyses included global SUVr, GM regional analyses and WM regional analyses. We compared uptake between FMT and PiB in cortical regions with both cerebellar crus (GM) as well as WM normalization and assessed WM regional uptake. To compare mean SUVr between PiB and FMT modalities we used Student's paired *t*-test. Group differences in SUVr between diagnostic groups, AD, eCN, and yCN, were assessed by using paired two-sample Student's *t*-tests. The purpose of the final set of regional analyses was to compare the performance of PIB and FMT ligands in terms of group-wise discrimination. We calculated the area under the receiver operating characteristic curve (AUROC) as a nonparametric measure of effect size (Acion et al., 2006) and calculated 95% confidence intervals for each AUROC estimate (Newcombe, 2006). We also directly tested group-wise discrimination as summarized by the AUROC for PiB versus FMT ligands (DeLong et al., 1988). All ROI-based analyses were performed using statistical software R (version 3.2.3).

## 3 Results

### 3.1 Subject demographics

Subject demographics and an overview of region findings in the groups are shown in Table 1.

**Table 1:** Subject demographics and imaging findings by diagnosis group.

Characteristic	AD	eCN	yCN
No. subjects	21	31	30
Age			
Mean (SD)	67 (9)	69 (6)	40 (6)
Median [IQR]	66 (62, 72)	68 (64, 72)	39 (34, 45)
Min, max	54 to 85	61 to 83	30 to 49
Male	11 (52%)	11 (35%)	12 (40%)
Global PiB SUVr (crus)			
Mean (SD)	2.9 (0.43)	1.5 (0.32)	1.2 (0.08)
Median [IQR]	2.92 (2.80, 3.15)	1.37 (1.31, 1.47)	1.19 (1.17, 1.21)
Min, max	1.8 to 3.6	1.2 to 2.5	1.1 to 1.6
Global FMT SUVr (crus)			
Mean (SD)	2.8 (0.45)	1.6 (0.29)	1.3 (0.09)
Median [IQR]	2.82 (2.61, 2.97)	1.47 (1.41, 1.56)	1.26 (1.24, 1.32)
Min, max	1.8 to 4.1	1.3 to 2.4	1.2 to 1.6
Global PiB SUVr (composite)			
Mean (SD)	1.8 (0.29)	0.9 (0.16)	0.8 (0.05)
Median [IQR]	1.82 (1.73, 1.99)	0.82 (0.78, 0.86)	0.76 (0.74, 0.80)
Min, max	1.1 to 2.3	0.7 to 1.5	0.7 to 0.9
Global FMT SUVr (composite)			
Mean (SD)	1.4 (0.22)	0.7 (0.14)	0.6 (0.05)
Median [IQR]	1.39 (1.28, 1.55)	0.69 (0.66, 0.74)	0.62 (0.60, 0.66)
Min, max	1.0 to 1.9	0.6 to 1.2	0.6 to 0.8
Days between PiB and FMT scans			
Median [IQR]	2 (1, 29)	2 (1, 10)	4 (2, 38)
Min, max	1 to 154	1 to 183	1 to 357

### 3.2 Voxel based analysis

**Fig. 1:**
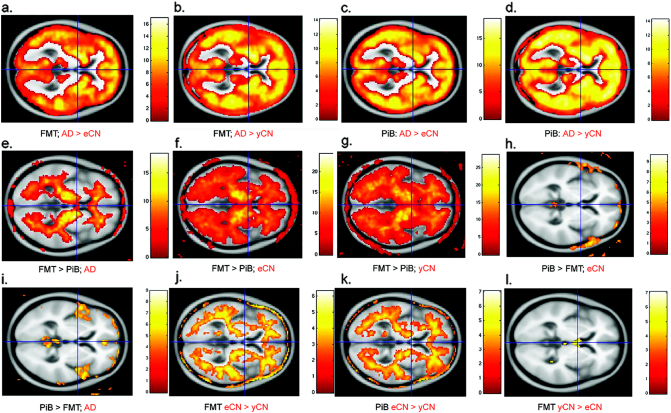
Voxel based comparisons of FMT and PiB in diagnostic groups. SPM findings are shown for whole brain quantitative differences by diagnostic group (in red; AD, eCN and yCN) and tracer (in black; PiB and FMT). Both FMT and PiB show greater signal throughout GM structures in AD vs. eCN or yCN (a–d). FMT has greater WM signal in any group vs. PiB (e–g) and also more grey matter uptake in CN (f and g). PiB did not have greater uptake than FMT in CN (h; eCN shown) and only slightly greater uptake in frontal regions in AD (i). Both FMT and PiB show more WM in eCN vs. yCN (j and k) while no greater uptake is seen in yCN vs. eCN (l; shown for FMT). All contrasts were false discovery rate (FDR) corrected at *p* < 0.05. (For interpretation of the references to color in this figure legend, the reader is referred to the web version of this article.)

On voxel-based analyses, both FMT and PiB showed greater GM uptake in AD vs. CN as expected (Fig. 1, a–d). Greater WM uptake was seen with FMT vs. PiB in all groups (Fig. 1, e–g). The greater uptake with FMT vs. PiB extended into the GM regions in both CN groups (Fig. 1, f and g). Frontal regions had modest increased uptake with PiB vs. FMT in AD but no increased uptake was seen in CN groups (Fig. 1, h and i). When comparing yCN to eCN, greater WM uptake was seen in eCN vs. yCN for both FMT and PiB (Fig. 1, e and f). Neither tracer showed greater uptake in yCN vs. eCN (l, eCN shown as an example with FMT).

### 3.3 Regional analysis of GM comparisons

**Fig. 2:**
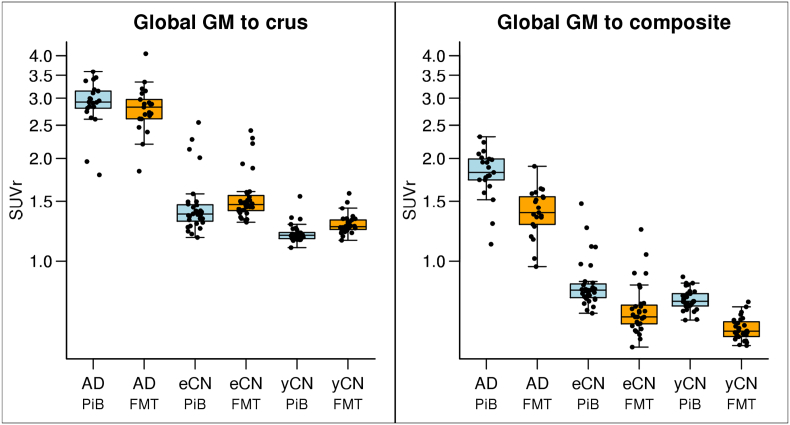
Global SUVr by diagnostic group (AD, eCN, yCN) and modality (PiB, FMT). Global SUVr are shown for each diagnostic group and show good group-wise separation between AD and CN groups for each modality. Normalization was performed using the crus (a) of the cerebellum and a composite region (b). The different normalization lowers all SUVr levels and changes the relationship of eCN and yCN between PiB and FMT such that FMT SUVr is greater in FMT in all CN with crus normalization but less with composite normalization.

**Fig. 3:**
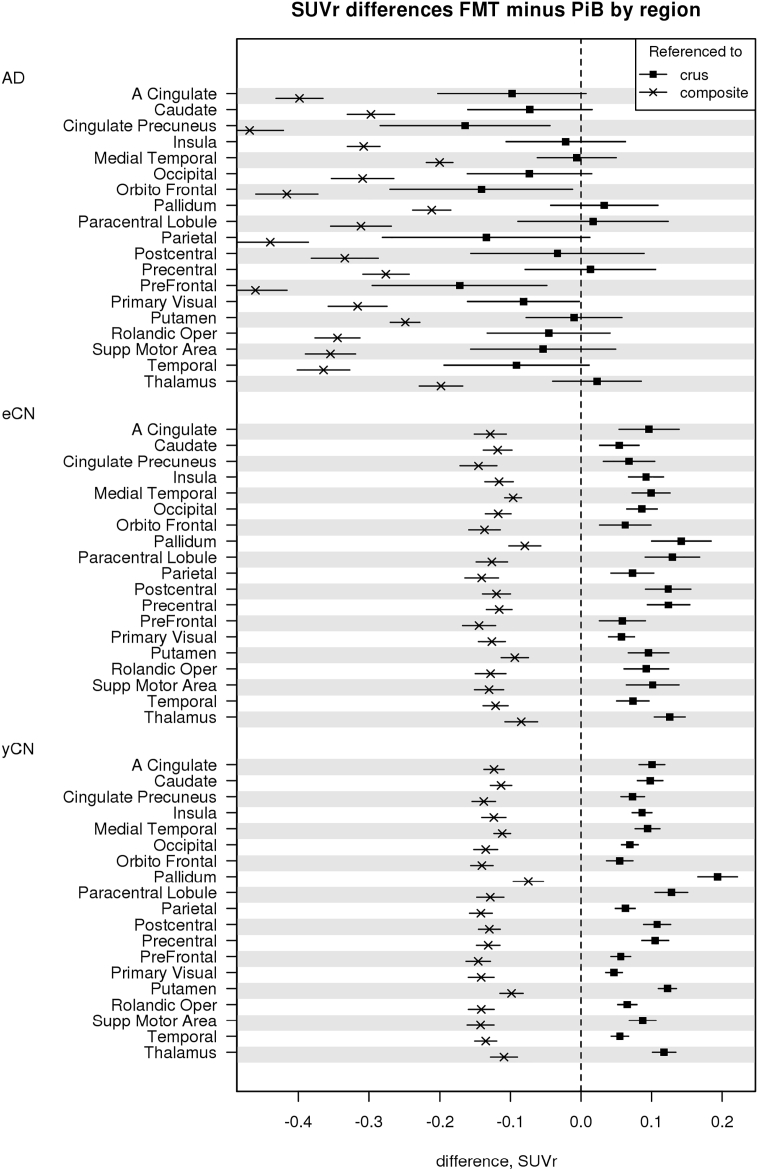
Differences in individual GM ROI SUVr seen in diagnostic groups. Mean SUVr differences (95% confidence interval (CI)) between FMT and PiB in GM regions for each patient group are shown as the differences in SUVr, FMT minus PiB, for each region. If the CI includes zero it is not significant, there is no significant difference between methods. In AD, when both are referenced to WM, FMT SUVr is less than PiB, reflecting more WM binding (from Fig. 2). Also in AD, when both are referenced to crus, there is a similar trend in most regions, and significant in some (e.g., posterior cingulate, orbitofrontal, prefrontal and primary visual) for lower FMT SUVr vs. PiB, likely reflecting less binding in the same individual GM regions with FMT. For eCN and yCN, FMT SUVr is less than PiB using composite white matter but reverses for crus normalization reflecting the greater WM uptake and greater regional GM uptake in FMT vs. PiB in CN.

Good separation of AD vs. CN groups was seen for both global FMT and PiB as shown in SUVr boxplots (Fig. 2). PiB and FMT global SUVr were similar in the AD group with crus normalization but FMT SUVr was less than PiB when WM composite was used for normalization. FMT tended to have greater SUVr in CN groups with crus normalization but reduced GM signal with WM composite region normalization. These findings in CN groups persisted even when amyloid positive CN participants (11 elderly CN and 1 young CN, all concordantly positive on FMT and PiB by visual analysis) were removed from the analysis (Inline Supplementary Fig. 2). In individual ROI analyses, significant AD vs. CN group-wise differences between PiB and FMT were observed (*p* < 0.001 for all regions). The differences within subject groups between regional FMT and PiB in AD were insignificant in select regions when using crus but were all significant with composite WM normalization (AD, Top of Fig. 3). Significant regional differences between FMT vs. PiB SUVr in CN were positive or negative depending on whether crus (positive) or WM (negative) normalization was used (Fig. 3). We also used 3-compartment PVC to assess the differences in FMT and PiB GM signal (when using crus normalization). Elevated GM FMT uptake vs PIB uptake was seen in yCN with similar trends in AD and eCN as compared to 2-compartment PVC (Inline Supplementary Figs. 4 and 5).

**Fig. 4:**
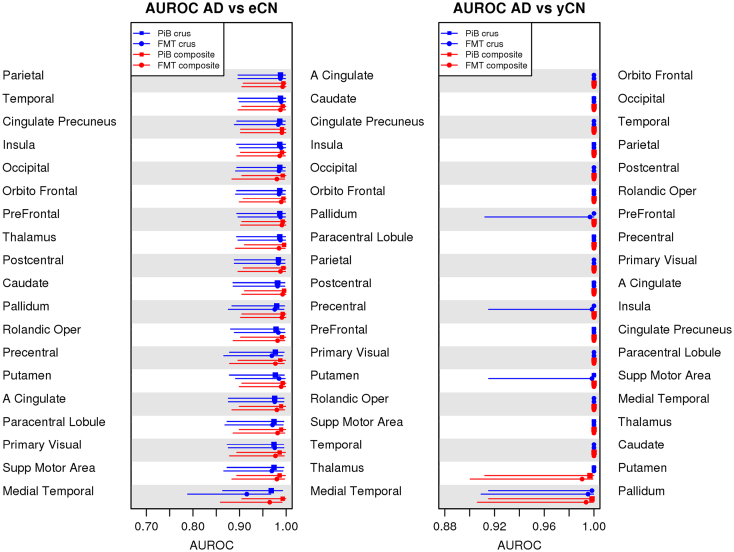
AUROC performance in individual GM ROIs. AUROC tables that compare the ability of WM composite and GM crus reference regions for both FMT and PiB to characterize diagnostic groups are shown. In eCN vs. yCN (right) a trend for lower accuracy with WM rather than with a crus reference regions is seen but no differences are seen in the other group comparisons (i.e., AD vs. CN).

AUROC as a measure of effect size was similar between FMT and PiB for all group pairings of AD and CN (Fig. 4). Differences were seen in discrimination between eCN and yCN groups with a trend for poorer discrimination when using WM normalization. This trend was seen with both PiB and FMT (Fig. 4, panel AUROC eCN vs. yCN, red lines).

### 3.4 Regional analysis of WM differences

**Fig. 5:**
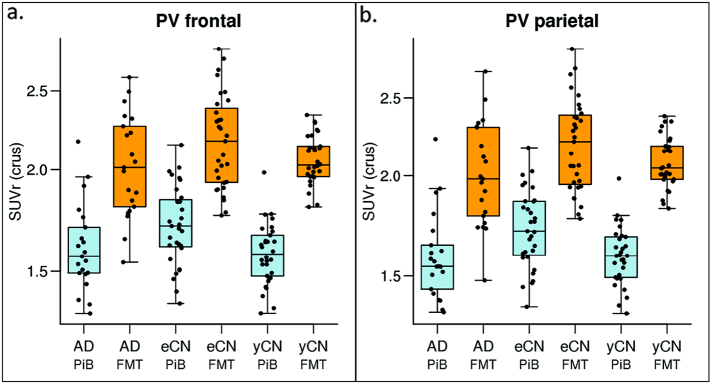
Regional WM group-wise SUVr findings. Boxplots of regional WM SUVr by diagnostic group (AD, eCN, yCN) and modality (PiB, blue, and FMT, orange) for periventricular frontal and parietal regions show that WM uptake with FMT is greater in all groups. WM uptake shows a trend for greater uptake in eCN vs. yCN for both tracers. (For interpretation of the references to color in this figure legend, the reader is referred to the web version of this article.)

**Fig. 6:**
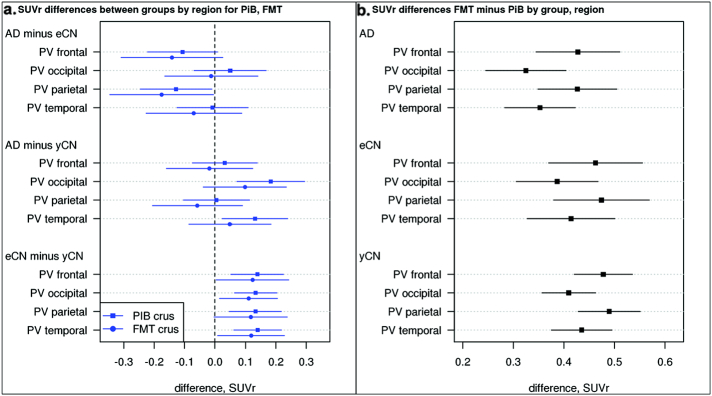
FMT and PiB Regional WM SUVr Differences. Mean SUVr differences (95% CI) between diagnostic groups in WM regions (a.) show reduced white matter uptake in AD vs. eCN primarily in the frontal and parietal periventricular (PV) WM but increased uptake in the occipital and temporal PV WM in AD vs. yCN. Results are shown for each individual tracer. If the CI does not include 0, then the diagnostic groups (i.e., AD v eCN) have significantly different uptake ratios according to a *t*-test. Positive differences in eCN vs. yCN are seen similar to voxel-based findings. B) Mean SUVr differences (95% CI) between PiB and FMT in GM regions. For example, the difference in AD for PV frontal region, FMT SUVr is significantly greater than PiB SUVr.

Regional WM SUVr were elevated when comparing FMT vs PiB in all groups (Fig. 5, shown for two WM regions). Subtraction of the WM SUVr (using only crus normalization) there is unexpectedly reduced *WM* SUVr in AD vs. eCN in two regions (frontal and parietal WM, Fig. 6, top left) with FMT and PiB. This difference is not seen in AD vs yCN suggesting the age-related increase in WM seen with both tracers is a possible cause. Indeed, FMT and PiB had greater WM accumulation in eCN vs. yCN (Fig. 6A, bottom). This corresponds to a similar finding noted above in the voxel-wise analysis (Fig. 1). There is a trend for relatively increased WM SUVr in AD compared to yCN participants with FMT and PiB. FMT demonstrated elevated WM uptake vs. PiB in all WM regions of all groups (Fig. 6b).

## 4 Discussion

The findings of this work demonstrate that amyloid PET tracers are not identical in their uptake characteristics and this has important implications. These characteristics are important to understand given that many amyloid tracers are available for research and clinical practice and various acquisition and analysis methods are used. In this work we demonstrate several important findings: first, we found quantitatively greater WM uptake with FMT vs. PiB in AD, yCN and eCN subjects; second, we found that FMT has greater GM uptake than PiB in yCN and eCN subjects; third we found an age relationship of WM uptake in both tracers, and forth, we demonstrated that these variations in tracer uptake can alter the tracer SUVr relationships in CN subjects.

We generally observed, by both voxel-based and regional analyses, greater WM uptake with FMT vs. PiB. We sampled several different WM ROIs and limited any signal spillover effects from GM into the WM regions by selecting periventricular WM regions. White matter uptake is considered nonspecific and has been shown to be nonsaturatable and largely attributable to specific WM kinetics (Fodero-Tavoletti et al., 2009). In a prior report comparing visual analysis of FMT and PiB in the same patients, higher uptake in the WM with FMT vs. PiB was reported (Hatashita et al., 2014). This resulted in a loss of grey/white matter distinction in positive cases for FMT compared to visually greater uptake in the GM vs. WM in PiB positive cases. These findings are supported by our data. Our data suggest that different quantitative approaches deserve a cautious approach given the WM uptake differences. We also found reduced WM SUVr in AD vs. eCN. This was not seen in the AD vs yCN, but given the age-related increase in WM uptake, the age-matched eCN group is the more appropriate comparison.

We have also shown that GM uptake is greater in FMT vs. PiB in CN participants (Fig. 1) and less than PiB in AD (Fig. 2). While our data and that of others (Hatashita et al., 2014) (Thurfjell et al., 2014) have shown that there is no significant difference in group-separation accuracy of AD and CN groups by FMT vs. PiB, other implications can be postulated. Possibilities for this increased GM uptake by FMT in CN include greater non-specific GM uptake, better detection of diffuse amyloid accumulation, or something yet to be understood. We would suspect that any nonspecific GM uptake would be reflected equally in GM and the reference region (if GM) and makes this possible cause less likely. Diffuse plaque has been shown to have less intense PiB-PET signal (Ikonomovic et al., 2008). Presumably FMT uptake in diffuse plaque would be similar and the increased GM uptake with FMT would not likely represent amyloid. It is not yet possible to understand the tracers' comparable sensitivities for identifying early or low-levels of amyloid plaque accumulation or the presence of diffuse plaque without autopsy correlation with both tracers. The comparative performance of the two tracers in such settings deserves further attention. Lastly, other image based effects like WM bleed-in to GM could influence this finding. We found that these differences persisted in yCN and had similar trends in eCN and AD after 3-comparment PVC, which should correct for this effect if bleed-in from white matter was the cause. While, the elevated uptake in GM with FMT in CN does not have any effect on group categorization, its effect on side-by-side performance with other tracers in serial analyses of patients when used to assess small changes in amyloidosis remains unknown and would be important to understand.

Changing SUVr relationships with FMT vs. PiB between subject groups depending on the normalization regions used probably due to WM uptake variations when combined with GM variations are of great interest. The SUVr relationships for these tracers reversed in CN when normalized to WM vs. crus. When combined with greater GM uptake in FMT vs. PiB in CN, the resulting implications can be important. These two variations, WM and GM, could in lead to confusing SUVr results if both tracers are used for serial evaluation of a single patient. For example, in a serial evaluation of a patient being evaluated for development of amyloid, if after an FMT scan using WM normalization, a PiB scan is performed a few years later also with WM normalization (in an attempt to maintain consistency of normalization), the patient may appear to have accumulated amyloid (due to WM effects, Fig. 3). If, on the other hand, the follow-up PiB scan analysis is performed with crus normalization, and the prior FMT is reanalyzed with crus normalization for consistency, the SUVr could appear lower (as GM uptake is lower with PiB in CN). While the design of most serial research trials may avoid these challenges by selecting only one amyloid tracer, and one method of analysis, eventual clinical use of amyloid PET imaging may be faced with the reality of several different amyloid PET tracers being performed in the same person over many years maybe at different institutions with different analytical preferences being employed. While PiB may never be performed clinically, other, less homologously-structured F-18 tracers may demonstrate similar differences. Similar inter-tracer comparisons will need to be done to assess differences between other amyloid tracers.

It has been recently proposed that some of these challenges could be mitigated by using a *Centiloid Scale* calculation (Klunk et al., 2015). Calculating *Centiloid* values and normalizing data in a *Centiloid Scale* for each scan is intended to provide translation of the amyloid PET data from various amyloid PET scans to a standardized scale with PiB PET. This is done by setting the mean of a young CN group to 0 and the mean of an AD group to 100 and translating all scans into this 0–100 scale. This has been proposed as a method to normalize across institutions using different PET radiotracers and different methods of quantitative analysis. The necessary requirement, however, is that a dataset with a site's particular choice of amyloid tracer be performed prospectively in a group of 25 test subjects for calibration against PiB. Identical image acquisition and processing described in the *Centiloid* paper are suggested. Such calibration datasets for different tracers are planned to be available on the GAAIN website (http://www.gaain.org/). Any methodological variation such as different region segmentation, normalization region or acquisition time, would require further validation to allow for the calibration. However, this process may be a challenge for typical clinical sites for the foreseeable future. In addition, the accuracy of this translation method to homogenize the performance of different amyloid PET tracers in longitudinal, serial imaging has not been verified.

Of great importance is our observation that both FMT and PiB had greater uptake in WM in eCN vs. yCN (Fig. 7). The mean age difference between the groups was 29 years. The GM uptake was also different but this most likely reflects the increased rate of amyloid positivity in eCN vs. yCN (Table 1). Periventricular WM ROIs were used to minimize any potential spill over of GM signal into WM. If the WM uptake changes with age, cross-sectional SUVr determinations using WM normalization across wide age ranges would need to be age corrected. These findings need to be confirmed for other amyloid tracers but are important to consider for group categorization across large age spans. We have recently shown in our longitudinal serial data over < 6 years that inclusion of WM regions for normalization can optimize longitudinal data (Schwarz et al., 2016). When comparing groups spanning many more years, age-dependent WM amyloid tracer binding may contraindicate such WM approaches. One could postulate that even in intra-individual amyloid assessments, the typical small yearly amyloid change that occurs, could be affected by small changes in the normalization regions values. These effects would have to be tested in longer ages spans with serial amyloid imaging than exist today. The cause of this WM age effect is nevertheless unknown. Correlation with MR findings may be helpful to assess WM nonspecific binding from other aging effects as possible causes in future investigations.

Weaknesses of this work include the use of only FMT and PiB. Florbetaben and PiB have been shown by others to have equivalent diagnostic performance in group-wise analysis of AD and CN subjects using cerebellar cortex as a reference region (Villemagne et al., 2012). Higher WM uptake was also reported with Florbetaben. In this prior report, no WM differences between CN and AD for Florbetaben vs. PiB were seen but the CN and AD participants were age matched. One additional interesting finding from Barthel, et al., was of lower Florbetaben SUVr in AD vs. CN in WM regions, such as cerebral WM, pons, and cerebellar WM (Barthel et al., 2011). This is in contrast to our FMT and PiB findings but demonstrates the complexity of using multiple amyloid tracers.

Weaknesses in this work include the lack of comparisons with other amyloid tracers and the lack of pathologic verification of the cases included. Assessments of WM uptake characteristics may differ for different amyloid tracers and would need to be evaluated independently. The eCN and yCN groups differ by 29 years and therefore one cannot infer the age effects in groups of different age differences. These studies were performed without full pharmacokinetic modeling and therefore the findings only show apparent binding. Nevertheless, these findings do reflect the challenges of late-uptake imaging as used by the majority of those in the field performing similar clinical studies and, therefore, the findings are widely generalizable. The strengths of this study include the direct comparison of two different amyloid tracers in the same subjects recruited prospectively and the use of both voxel-based and ROI-based quantitative analyses.

## 5 Conclusion

Flutemetamol and PiB show similar topographical GM uptake distributions in AD dementia and CN participants. Both imaging tracers have comparable group discrimination. Differences in WM accumulation (FMT > PiB) between the two amyloid tracers suggest that quantitative differences will be apparent when using WM or GM as reference regions. WM uptake is higher in older people in both tracers. These variations could result in artifactual quantitative differences in amyloid scans in serial imaging. Clinical use of several amyloid tracers in the same patient will have many challenges that need to be carefully considered.

## Funding

This work was supported by GE Healthcare Inc., NIH grants P50 AG16574, U01 AG06786, R01 AG11378, RO1 AG041851, the Dekelboum Foundation and the Robert H. and Clarice Smith and Abigail Van Buren Alzheimer's Disease Research Program of the Mayo Foundation.

## Potential conflicts of interest

Dr. Lowe is a consultant for Bayer Pharma, Piramal Imaging, and receives research support from GE Healthcare, AVID Radiopharmaceuticals, the NIH, the Dekelboum Family Foundation, and the Liston Family Foundation.

Ms. Lund, no conflict of interest.

Dr. Knopman serves on a Data Safety Monitoring Board for Lundbeck Pharmaceuticals and for the DIAN study; is a clinical investigator for Biogen, TauRX Pharmaceuticals, Lilly Pharmaceuticals and the Alzheimer's Disease Cooperative Study; and receives research support from the NIH.

Mr. Senjem owns stock and/or stock options in the following medical companies, unrelated to the present study: Celgene Corporation, Inovio Pharmaceuticals, Medtronic, Inc., Parexel International Corporation, and Gilead Sciences, Inc.

Dr. Gunter, no conflict of interest.

Dr. Schwarz, no conflict of interest.

Dr. Kemp, no conflict of interest.

Dr. Jack serves on scientific advisory boards for Siemens; receives research support from the NIH/NIA, and the Alexander Family Alzheimer's Disease Research Professorship of the Mayo Foundation; and holds stock in Johnson & Johnson.

Dr. Petersen serves on scientific advisory boards for Pfizer, Inc., Janssen Alzheimer Immunotherapy, Elan Pharmaceuticals, and GE Healthcare; receives royalties from the publication of *Mild Cognitive Impairment* (Oxford University Press, 2003); and receives research support from the NIH/NIA.

## Appendix A Supplementary data

Supplementary figures Image 1
