# Supplementary material for: Comparison of [18F]Flutemetamol and [11C]Pittsburgh Compound-B in cognitively normal young, cognitively normal elderly, and Alzheimer's disease dementia individuals
Source: Neuroimage Clin. 2017 Aug 14;16:295–302. doi: 10.1016/j.nicl.2017.08.011 (PMC5565786; doi:10.1016/j.nicl.2017.08.011)
Supplement: Supplementary file 1 — Supplementary figures [file mmc1.docx]

**Inline Supplementary Figures with Tltles and Legends.**


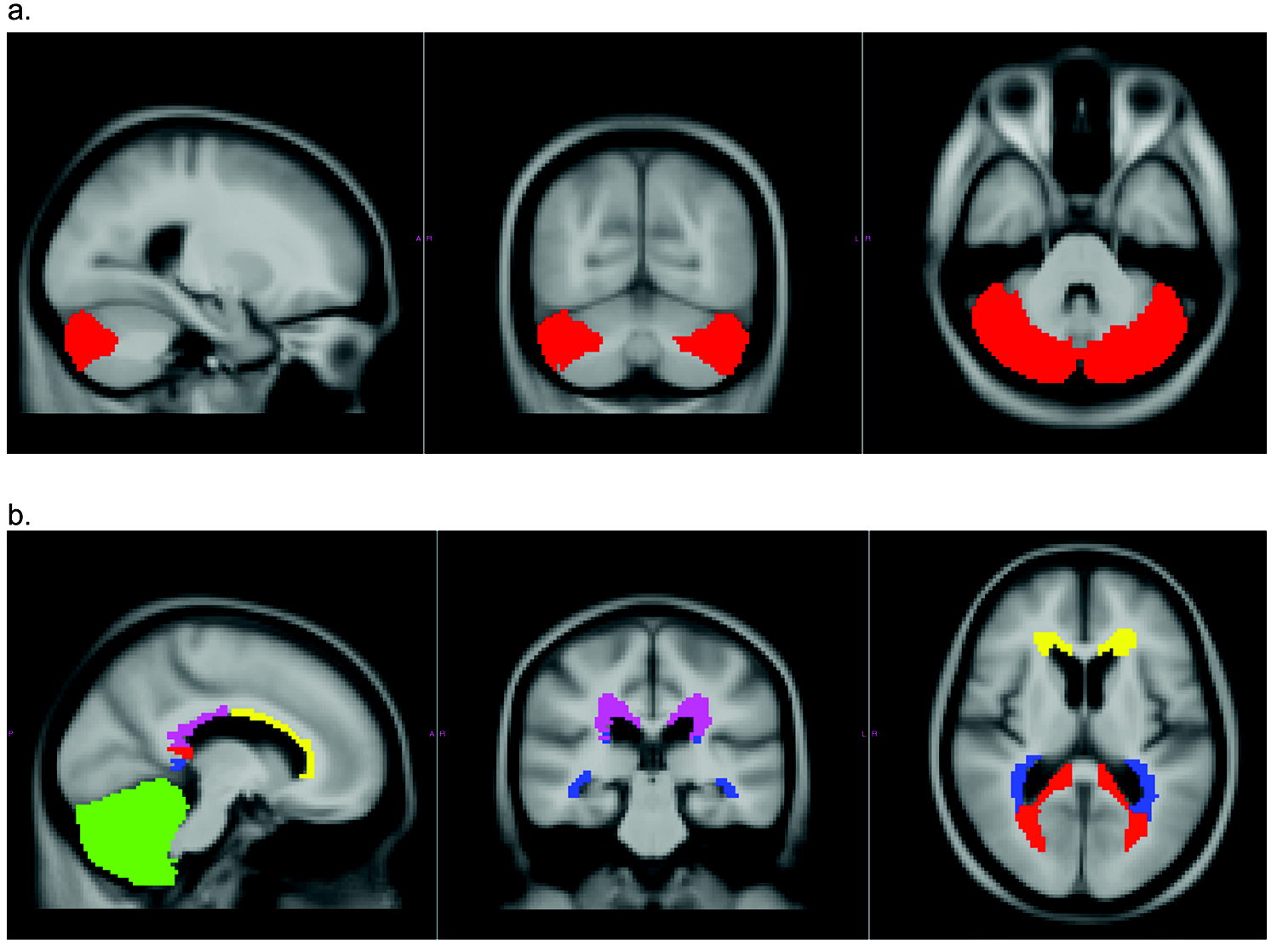


**Inline Supplementary Figure 1. Normalization regions.**

**Inline Supplementary Figure 1.**The GM regions of the cerebellum used for normalization and named crus 1 and crus 2 in the AAL atlas are shown in red in the bilateral cerebellar lobes in orthogonal views superimposed on a temple MRI (a). Periventricular (PV) WM regions used are demonstrated in similar 3 orthogonal views (b) with the PV frontal shown in yellow, the PV temporal shown in blue, PV parietal shown in purple and PV occipital shown in red. The whole cerebellum is shown in green. The composite region is made up of the WM regions shown and the whole cerebellum ROI (all of the regions shown in part “b”).


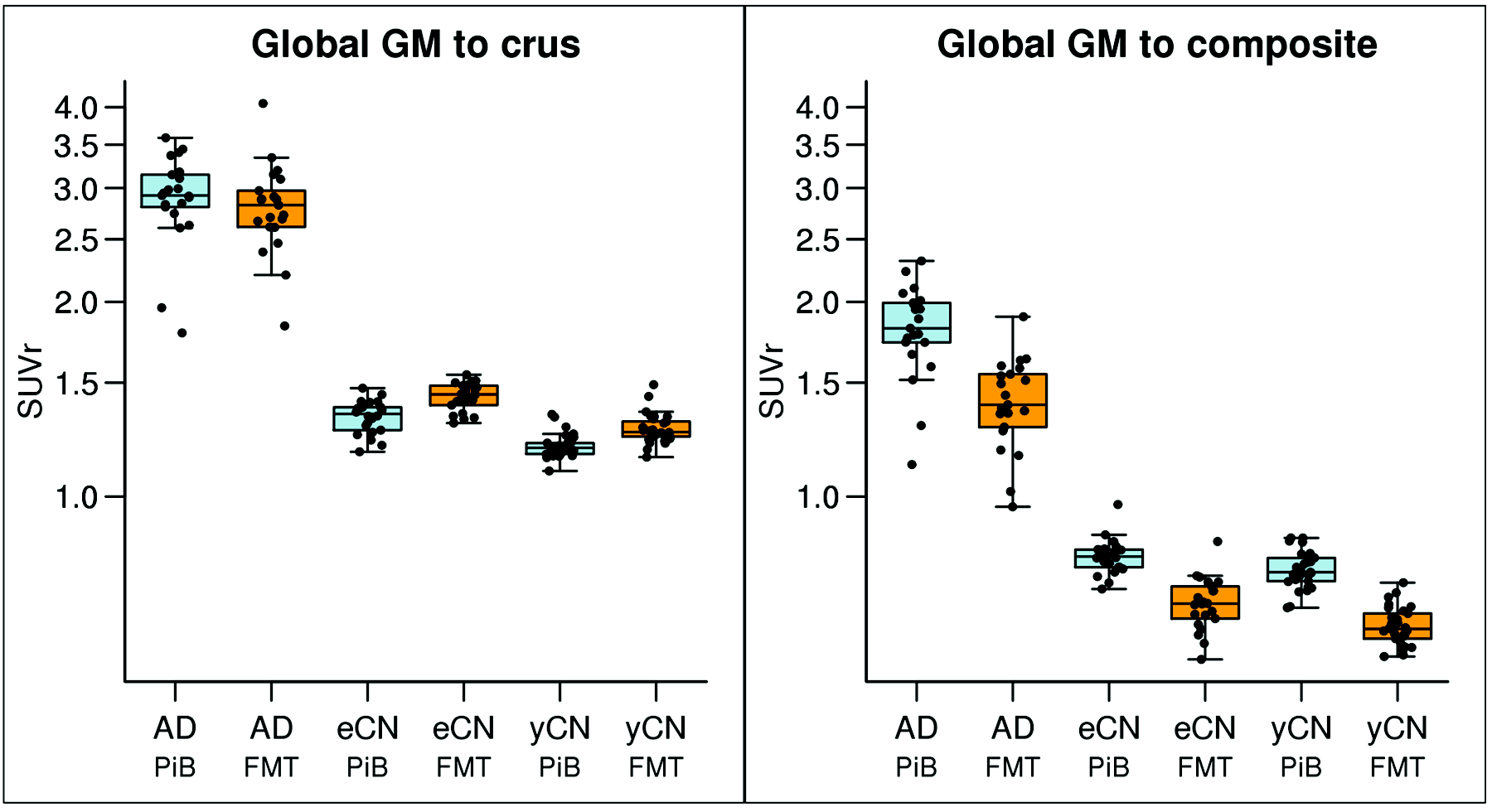


**Inline Supplementary Figure 2.** **Global SUVr by Diagnostic Group (AD, eCN, yCN) and Modality (PiB, FMT) Excluding Amyloid Positive CN Participants.**

**Inline Supplementary Figure 2**. Global SUVr are shown for each diagnostic group and show good group-wise separation between AD and CN groups for each modality after amyloid positive CN participants have been removed. Normalization was performed using the crus (a) of the cerebellum and a composite white matter region (b). The different normalization lowers all SUVr levels and changes the relationship of eCN and yCN between PiB and FMT such that FMT SUVr is greater in FMT in all CN with crus normalization but less with composite normalization.


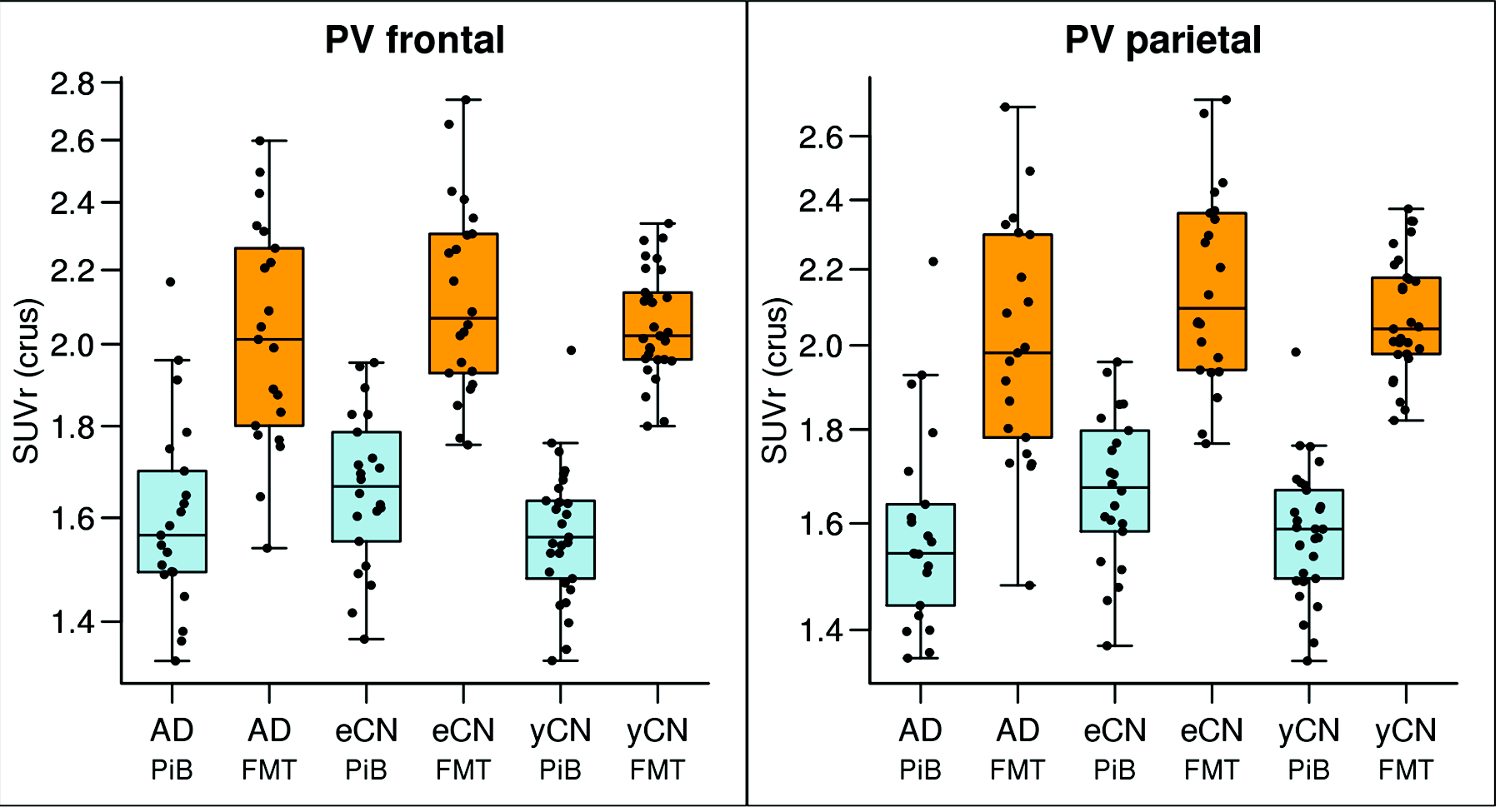


**Inline Supplementary Figure 3. Regional WM Group-Wise SUVr Findings with Amyloid Positive CN Participants Removed.**

**Inline Supplementary Figure 3.** Boxplots of regional WM SUVr by diagnostic group (AD, eCN, yCN) and modality (PiB, blue, and FMT, orange) for periventricular frontal and parietal regions show that WM uptake with FMT is greater in all groups. WM uptake shows a trend for greater uptake in eCN vs. yCN for both tracers.


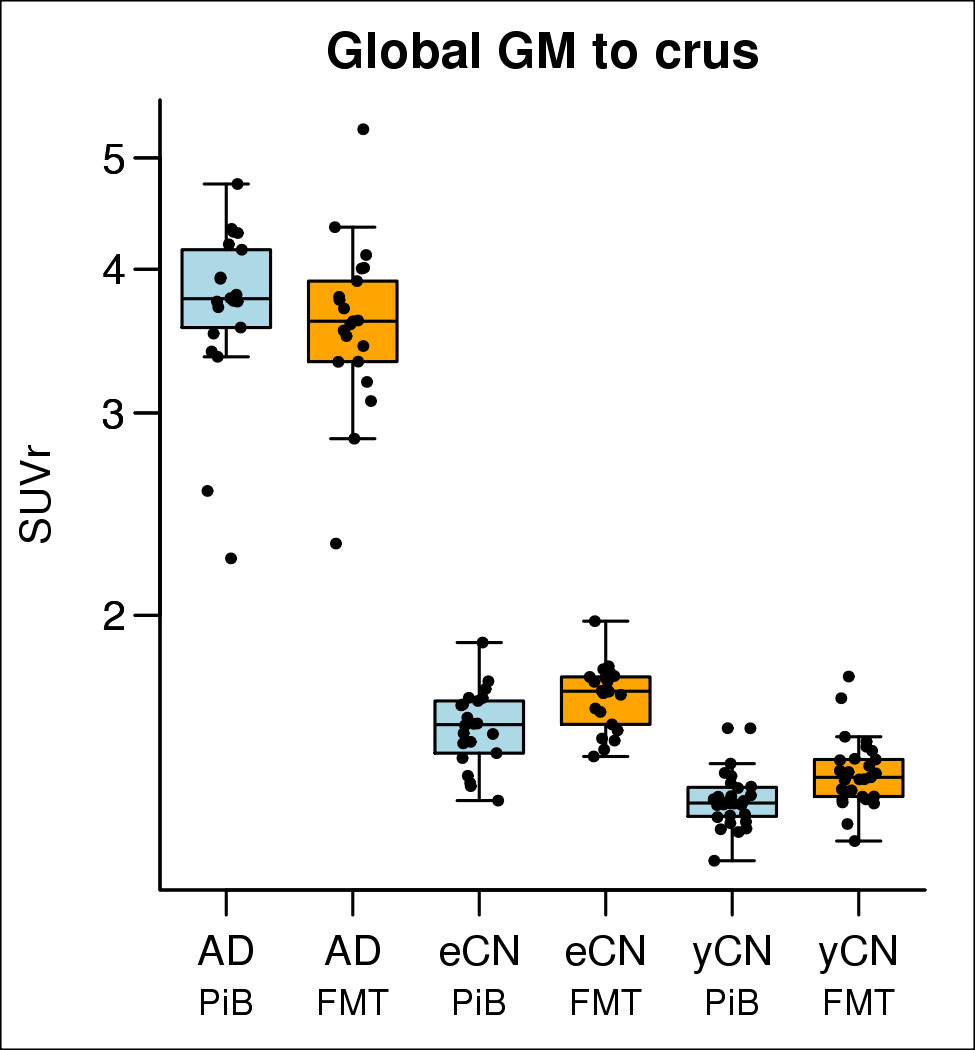


**Inline Supplementary Figure 4.** **Global SUVr by Diagnostic Group (AD, eCN, yCN) and Modality (PiB, FMT) with 3-Compartment Partial Volume Correction.**

**Inline Supplementary Figure 4.** The SUVr levels are greater for FMT vs. PiB in eCN and yCN, similar to that of Figure 2 when using 3-Compartment Partial Volume Correction (PVC-3). Normalization was performed using the crus.


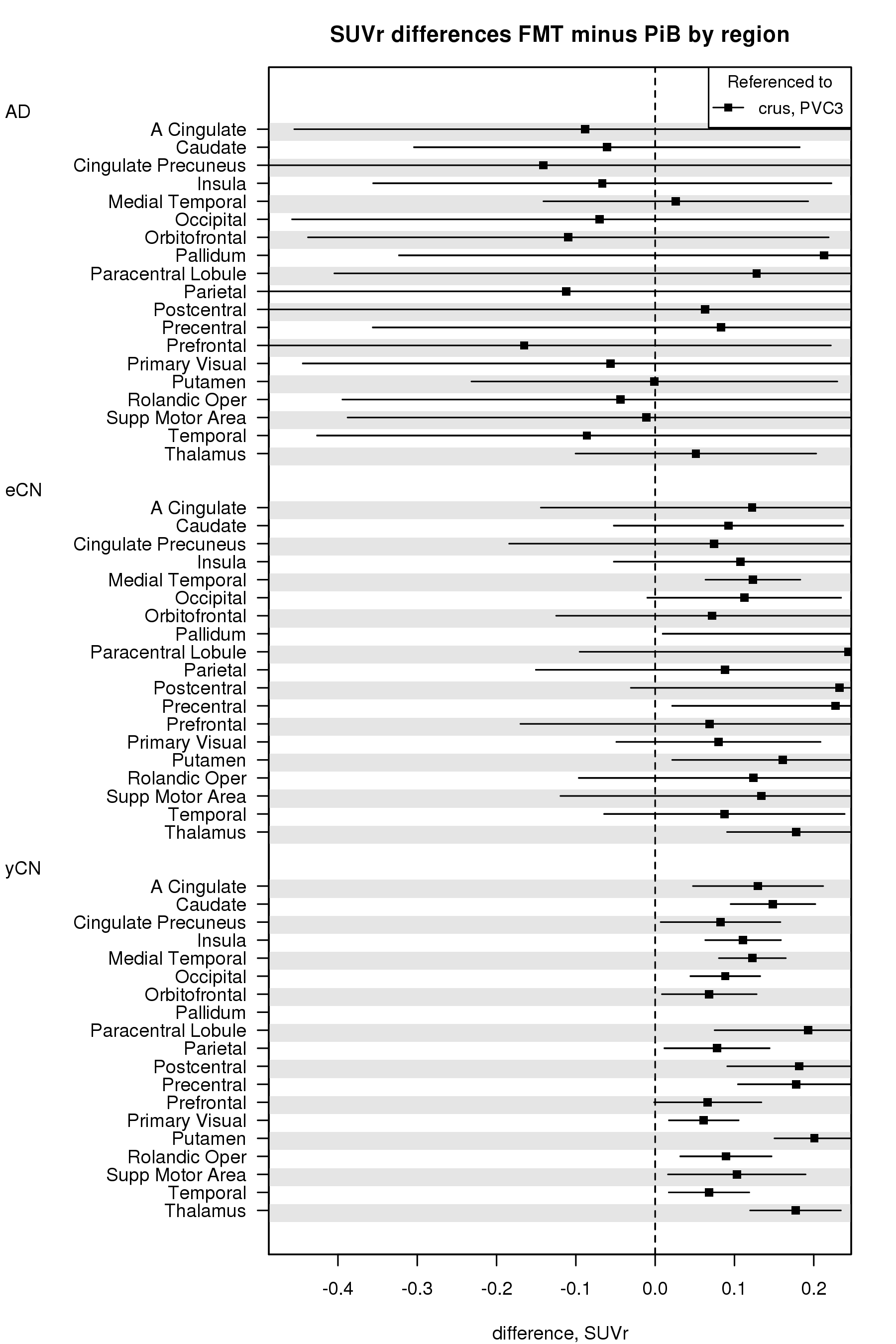


**Inline Supplementary Figure 5.** **Differences in Individual GM ROI SUVr Seen in Diagnostic Groups using 3-Compartment Partial Volume Correction.**

**Inline Supplementary Figure 5.** Mean SUVr differences (95% confidence interval (CI)) between FMT and PiB in GM regions for each patient group are shown as the differences in SUVr, FMT minus PiB, for each region when using 3-compartment partial volume correction (PVC-3). If the CI includes zero it is not significant and there is no significant difference between methods for a region. The reference area is the crus. For yCN, FMT SUVr is greater than PiB in all regions and a similar trend is seen in eCN. There is also a trend for greater PiB vs FMT in AD. While the confidence intervals are larger as compared to 2-compartment partial volume correction (Figure 3), likely to due the larger SUVr ranges produced with PVC-3, the trends are the same.
